# A success story: HIV prevention for injection drug users in Rhode Island

**DOI:** 10.1186/1747-597X-1-34

**Published:** 2006-12-04

**Authors:** Curt G Beckwith, Carla C Moreira, Hesham M Aboshady, Nickolas Zaller, Josiah D Rich, Timothy P Flanigan

**Affiliations:** 1 Brown Medical School, Department of Medicine, The Miriam Hospital, 164 Summit Ave, Providence, Rhode Island 02906, USA; 2 Rhode Island Department of Health, 3 Capitol Hill, Providence, Rhode Island, 02908, USA

## Abstract

**Background:**

New HIV diagnoses related to injection drug use (IDU) have declined in the United States. Access to clean syringes and decreasing HIV transmission among injection drug users have been HIV prevention priorities of the Rhode Island (RI) HIV community. To examine trends in IDU-related new HIV diagnoses in RI, we performed a retrospective analysis of new HIV diagnoses according to HIV risk factor from 1990–2003.

**Results:**

There has been an 80% absolute reduction in IDU-related new HIV diagnoses in RI coincident with IDU-specific prevention efforts.

**Conclusion:**

There has been a greater decline in IDU-related new HIV diagnoses in Rhode Island compared to national data reported by the Centers for Disease Control and Prevention. We hypothesize that this dramatic decline in Rhode Island is related to extensive HIV prevention efforts targeting IDUs. Further research is needed to examine the impact of specific HIV prevention interventions for injection drug users.

## Background

Injection drug-use has been a major mode of HIV transmission since the beginning of the HIV epidemic. However, in 2003, the Centers for Disease Control and Prevention (CDC) reported a 42% decline in HIV diagnoses among injection drug users (IDUs) from 1994–2000 [1]. More recent surveillance data demonstrated a 4.1% decline in HIV diagnoses among IDUs from 2001–2004 [2]. Recent studies in Baltimore and New York City have documented a decline in HIV incidence among injection drug users [3,4]. Factors that may contribute to the observed decline in HIV diagnoses related to IDU nationally include improved syringe access, improved education and harm reduction efforts targeting IDUs, the introduction of highly active antiretroviral therapy for the treatment of HIV infection, and the decline may represent the natural evolution of the HIV epidemic as the number of susceptible IDUs shrinks. As demonstrated in the study by Des Jarlais et al. in New York City, access to clean syringes may impact HIV incidence among IDUs [4]. However, the ability of IDUs to obtain clean syringes varies widely across the United States, largely based on the availability of syringe exchange programs and the ability to legally purchase syringes via pharmacies [5].

In 1989, 50% of HIV cases in Rhode Island were associated with injection drug use [6]. In an effort to decrease transmission of HIV between IDUs, prevention interventions including syringe exchange programs, syringe prescription programs, and legalization of syringe purchase at pharmacies were developed and implemented in Rhode Island from 1995–2000 [7-9]. The objective of this study was to examine trends in new HIV diagnoses related to injection drug use and other modes of transmission in Rhode Island from 1990–2003, the time period during which these IDU-related prevention interventions were implemented. Trends were examined retrospectively by reviewing the Rhode Island Department of Health HIV database (State database) and the Miriam Hospital Immunology Center database (Clinic database). We report a dramatic decline in the number of new HIV diagnoses attributed to injection drug use in Rhode Island coincident with the implementation of multiple HIV prevention interventions targeting IDUs.

## Results

**Figure 1:**
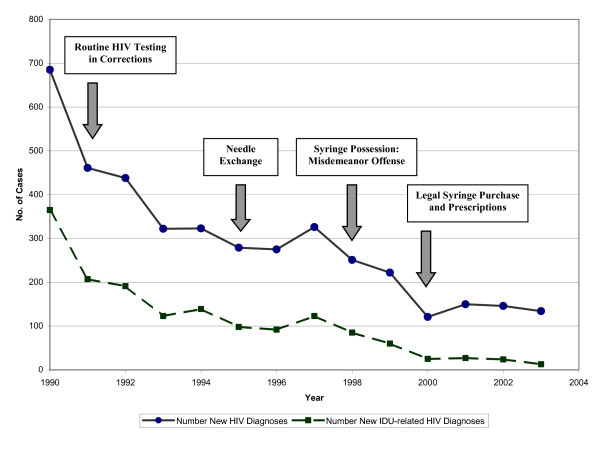
**Newly Diagnosed Cases of HIV Reported to the Rhode Island Department of Health by Year of Diagnosis**. The number of newly diagnosed cases of HIV in Rhode Island and the number of new HIV diagnoses related to injection drug use from 1990–2003. The implementation of specific HIV prevention interventions targeting injection drug users is noted in boxes.

According to the State database, which received HIV reports anonymously from 1990–1999 and then via unique identifier from 2000–2003, the number of new HIV diagnoses declined from 685 (78% male, 22% female) in 1990 to 134 in 2003 (77% male, 23% female). The number of IDU-related new HIV diagnoses declined from 365 in 1990 to 13 in 2003 (See Figure 1). The percentage of IDU-related new diagnoses in 1990 was 53% and this declined to 9.7% in 2003, an absolute reduction of 81%. Over the same time period, the percentage of new diagnoses related to MSM transmission and heterosexual transmission increased from 16% to 34% and from 4% to 19%, respectively.

According to the Clinic database, the number of newly diagnosed persons entering care remained relatively stable with 58 persons entering care in 1990 and 65 persons in 2003 (mean number of patients entering care per year = 53). The percentage of IDU-related newly diagnosed patients establishing care at the Clinic declined from 38% in 1990 to 5% in 2003, an absolute reduction of 87%. The percentage of new diagnoses related to MSM and heterosexual transmission increased from 21% to 31% and from 24% to 55%, respectively. The percentage of IDU-related new HIV diagnoses declined from 26% to 5% in males; 12% to 0% in females; 14% to 2% among Whites; 21% to 0% among Blacks; and increased from 2% to 3% among Hispanics.

## Conclusion

HIV infections secondary to injection drug-use have declined across the United States. The reasons for this decline are speculative however prevention programs targeting IDUs have likely contributed. Nevertheless, close to 4000 new cases of HIV/AIDS due to injection drug use were reported in 2004 suggesting that continued prevention efforts are necessary [2]. Unfortunately, many states still have strict laws and regulations prohibiting the possession of syringes contributing to syringe reuse and sharing among IDUs. Prevention efforts targeting IDUs have been a priority in Rhode Island and have been implemented through a multi-faceted approach over time. In 1994, Rhode Island authorized the opening of a syringe exchange program in Providence. In 1998, the state legislature reduced the penalty for possession of syringes from a felony to a misdemeanor and in 2000, Rhode Island passed legislation completely legalizing the sale of non-prescription syringes by pharmacists.

The correctional system has also been an important site for HIV prevention efforts targeting IDUs in Rhode Island. The state correctional facility has been conducting routine voluntary HIV testing upon entrance since 1991 and over 90% of all inmates are tested on incarceration [10]. Inmates who are identified as HIV-positive are linked to HIV medical care and other programs such as Project Bridge, a Ryan White CARE (Comprehensive AIDS Resources Emergency) Act Special Project of National Significance project aimed at maintaining continuity of medical care upon release from the correctional setting [11]. This program includes substance use treatment including linkage to methadone maintenance treatment in the community and intensive case management services for HIV-positive ex-offenders. Furthermore, in Rhode Island, there are six methadone maintenance treatment programs with 13 sites making this a widely available opiate replacement option for IDUs.

There are limitations to this analysis. The decrease in IDU-related new HIV diagnoses could be due to factors other than the harm reduction programs described. For instance, it is possible that the observed reduction is a reflection of a finite population of IDUs in Rhode Island being exposed to a new infectious disease to which they are susceptible (in this case, HIV infection). Since the size of the IDU population is relatively stagnant, the number of persons susceptible to HIV declines as more persons become infected. The introduction of highly active antiretroviral therapy in the late 1990's could have also led to decreased transmissibility of HIV between IDU's which may have led to a decrease in IDU-related HIV incidence. Finally, the change in HIV reporting method in Rhode Island from anonymous to unique identifier reporting in 2000 could have also contributed to the observed decline. With anonymous testing, IDU's who are HIV-positive would have been reported to the state anonymously each time testing was completed. Repeat testing is commonplace in the correctional system. Once unique identifier reporting was initiated, the potential for duplicate reporting would have been eliminated.

Nevertheless, our analysis reveals a greater than 80% decline in the annual percentage of new HIV cases in the state attributable to IDU over a 14-year time period. This decline is twice as great as the decline observed by the CDC in states with name-based HIV reporting. We hypothesize the comprehensive HIV prevention efforts targeting IDUs in Rhode Island are at least partially responsible for this dramatic decline although direct causality cannot be determined from this study. Further research is needed including comparisons of cities and states with and without prevention interventions to determine the relative contribution of these programs in decreasing the number of IDU-related new HIV infections. In addition, studies to investigate the effectiveness of individual prevention interventions are needed to guide future program implementation.

## Methods

A retrospective analysis of two databases was performed examining new HIV diagnoses according to risk category [IDU, men who have sex with men (MSM), and heterosexual] from 1990–2003. The data was obtained from the Rhode Island Department of Health (State database) and from The Miriam Hospital Immunology Center (Clinic database), which is the largest outpatient HIV clinic in Rhode Island. When possible, new HIV diagnoses were stratified by gender and race. This study was approved by The Miriam Hospital Institutional Review Board.

## Competing interests

The author(s) declare that they have no competing interests.

## Authors' contributions

CB conceived of the study and participated in its design, data interpretation, and drafting of the final manuscript. CM participated in data collection and interpretation. HA participated in data collection and interpretation. NZ participated in drafting of the final manuscript. JR participated in drafting of the final manuscript. TF participated in the conception of the study, data interpretation, and drafting of the final manuscript. All authors read and approved the final manuscript.
